# Interpretable Machine Learning Models to Predict the Resistance of Breast Cancer Patients to Doxorubicin from Their microRNA Profiles

**DOI:** 10.1002/advs.202201501

**Published:** 2022-07-03

**Authors:** Adeolu Z. Ogunleye, Chayanit Piyawajanusorn, Anthony Gonçalves, Ghita Ghislat, Pedro J. Ballester

**Affiliations:** ^1^ Cancer Research Center of Marseille (CRCM) INSERM U1068 Marseille F‐13009 France; ^2^ Cancer Research Center of Marseille (CRCM) Institut Paoli‐Calmettes Marseille F‐13009 France; ^3^ Cancer Research Center of Marseille (CRCM) Aix‐Marseille Université Marseille F‐13284 France; ^4^ Cancer Research Center of Marseille (CRCM) CNRS UMR7258 Marseille F‐13009 France; ^5^ Department of Bioengineering Imperial College London London SW7 2AZ UK

**Keywords:** artificial intelligence, machine learning, multiomics, precision oncology, tumor profiling

## Abstract

Doxorubicin is a common treatment for breast cancer. However, not all patients respond to this drug, which sometimes causes life‐threatening side effects. Accurately anticipating doxorubicin‐resistant patients would therefore permit to spare them this risk while considering alternative treatments without delay. Stratifying patients based on molecular markers in their pretreatment tumors is a promising approach to advance toward this ambitious goal, but single‐gene gene markers such as HER2 expression have not shown to be sufficiently predictive. The recent availability of matched doxorubicin‐response and diverse molecular profiles across breast cancer patients permits now analysis at a much larger scale. 16 machine learning algorithms and 8 molecular profiles are systematically evaluated on the same cohort of patients. Only 2 of the 128 resulting models are substantially predictive, showing that they can be easily missed by a standard‐scale analysis. The best model is classification and regression tree (CART) nonlinearly combining 4 selected miRNA isoforms to predict doxorubicin response (median Matthew correlation coefficient (MCC) and area under the curve (AUC) of 0.56 and 0.80, respectively). By contrast, HER2 expression is significantly less predictive (median MCC and AUC of 0.14 and 0.57, respectively). As the predictive accuracy of this CART model increases with larger training sets, its update with future data should result in even better accuracy.

## Graphical abstract

How well can the response of breast cancer patients to doxorubicin‐containing treatments be currently predicted? An unusually broad analysis (8 molecular profiles and 16 machine learning algorithms) reveals that only 2 of the 128 resulting classifiers are predictive (MCC > 0.3). Therefore, considering fewer algorithms and/or fewer profiles could easily result in not being able to anticipate doxorubicin response.

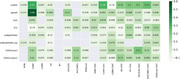

## 1 Introduction

Breast cancer (BC) has the highest global incidence and mortality rate amongst all cancer types affecting women.^[^ ^1^, ^2^ ^]^ In 2020, BC rose above lung cancer to become the most frequently diagnosed cancer worldwide,^[^ ^1^ ^]^ with over 2 million new cases recorded (11.7% of all reported new cases) and over 600 000 deaths (6.9% of the overall cancer deaths recorded). Doxorubicin is an intravenous chemotherapy, within the anthracycline class of drugs, used in both the early and the advanced setting of various cancer types, including BC.^[^ ^3^, ^4^ ^]^ Doxorubicin works essentially by intercalating between neighboring DNA base pairs.^[^ ^5^, ^6^ ^]^ The resulting doxorubicin–DNA complex inhibits topoisomerase II activity, which subsequently disrupts DNA replication and transcription, leading to both cytotoxic and apoptotic cell death.^[^ ^7^, ^8^ ^]^ Unfortunately, primary resistance to treatment is common across cancer types and drugs.^[^ ^9^, ^10^, ^11^, ^12^ ^]^ Such de novo resistance is also commonly observed with doxorubicin‐containing treatments in BC patients.^[^ ^13^ ^]^

A central goal of precision oncology is to anticipate which patients will be resistant to a given drug.^[^ ^14^, ^15^ ^]^ This anticipation would result in the identified patients receiving without delay alternative treatments more likely to stop cancer progression. In the case of doxorubicin, which may cause cardiotoxicity,^[^ ^7^, ^16^, ^17^ ^]^ such predictors would also be helpful to avoid resistant‐predicted patients taking unnecessary risks. Historically, this goal has been approached by searching for a single‐gene marker, which is a molecular feature able to distinguish between sensitive and resistant tumors to the treatment. While many efforts have employed preclinical data with this purpose,^[^ ^18^, ^19^, ^20^, ^21^ ^]^ much less abundant clinical datasets are by definition the most relevant for patients.^[^ ^14^ ^]^ An example of the latter clinical studies is one where high HER2 expression in BC patient tumors was reported to be predictive of patient response to anthracyclines, including doxorubicin.^[^ ^22^ ^]^ A major limitation, however, is that many single‐gene markers only provide modest predictive accuracy restricted to a very small fraction of patients.^[^ ^15^ ^]^ As an example, 16% of EGFR‐mutant non‐small‐cell lung cancer (NSCLC) patients were found to respond to Erlotinib, but the prevalence of this mutation was low and 7% of EGFR‐WT NSCLC patients also responded to this drug.^[^ ^23^ ^]^ Thus, at least in this cohort, the Matthew correlation coefficient (MCC) of this FDA‐approved single‐gene marker is just 0.11,^[^ ^24^ ^],^ i.e., slightly more predictive than random guessing. These practical limitations of single‐gene markers may be due to biological reasons (e.g., patient response to a drug is the outcome of a complex multifactorial process, which is often poorly anticipated by the status of a single gene), but also technical reasons (e.g., using metrics that can strongly overestimate predictive performance in the common scenario where there is a class imbalance in the data).^[^ ^25^, ^26^ ^]^ Multigene expression signatures of drug response are also a common approach, but these are limited by neglecting epistatic effects and profiles other than messenger RNA (mRNA) expression.^[^ ^27^, ^28^, ^29^, ^30^ ^]^

These limitations are now being overcome by machine learning (ML), which can generate computational models exploiting multiple pretreatment features of patients to predict their response to that drug treatment. There are many of such computational studies using pharmaco‐omic data from preclinical models,^[^ ^31^ ^]^ especially data from cancer cell lines^[^ ^32^, ^33^, ^34^, ^35^ ^]^ but also data from primary tumor cultures^[^ ^36^, ^37^ ^]^ or from patient‐derived xenografts.^[^ ^24^, ^38^ ^]^ When systematically and directly compared to single‐gene markers, multigene ML models mostly offer higher MCC and almost always much higher recall.^[^ ^39^, ^40^ ^]^ However, while some progress on leveraging preclinical data with this purpose has been made,^[^ ^24^, ^41^, ^42^, ^43^, ^44^, ^45^, ^46^, ^47^ ^]^ preclinical models still tend to struggle to predict drug response in patients with a useful level of accuracy.^[^ ^48^, ^49^ ^]^

ML models exploiting clinical data are hence attractive in this context. However, such studies also have their challenges, e.g., the scarcity of suitable datasets and the need for substantial curation before these datasets can be used for this purpose. For instance, a patient tumor's molecular features are likely to be altered upon drug treatment.^[^ ^50^, ^51^ ^]^ Therefore, as these are unlikely to represent the pretreatment molecular features of the tumor faithfully, one aim of curation is to discard tumors that were profiled after the drug is administered (otherwise, these noisy data instances could degrade model performance). Following such curation, Ding et al. built binary classifiers for six drug‐cancer type binomials,^[^ ^52^ ^]^ each exploiting four molecular profiles: copy number alteration, DNA methylation, mRNA, and microRNA (miRNA). An elastic net with bootstrapping was used for each profile to select the most predictive molecular features, and a final ensemble classifier was built based on these features. Using fivefold cross‐validation (CV) predictions of drug treatment response, the area under the curve (AUC) of the receiver operating characteristic (ROC), hereafter AUC for short, was above random‐guess level in 4 of the 6 binomials in at least one of the considered profiles. In Bomane et al.,^[^ ^53^ ^]^ our lab focused on a single drug‐cancer type binomial, paclitaxel‐BC, but expanded the analysis to 6 profiles and 10 ML algorithms. Interestingly, miRNA and DNA methylation profiles were revealed to be highly predictive for this binomial. However, the latter only occurred with two algorithms: classification and regression tree (CART) and, to a lesser extent, extreme gradient boosting (XGBoost) integrated with feature selection. Overall, there are still few studies on the application of ML to predict drug treatment response, in part due to the scarcity of suitable clinical samples for a given drug‐cancer type binomial, although its application to predict other patient outcomes requiring less curation has received more attention (e.g., prognosis).^[^ ^54^ ^]^

**Figure 1:**
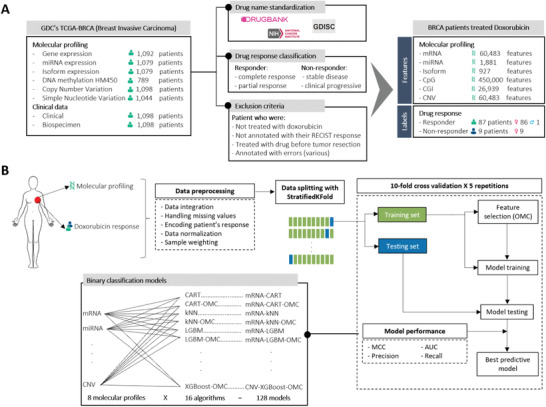
Schematic representation of the development of supervised learning models to predict patient response to doxorubicin. A) Multiomics datasets, including molecular profiles of patient tumors generated with high‐throughput technologies such as RNA‐Sequencing, miRNA‐Sequencing or DNA methylation array, were retrieved from the NCI GDC data repository. The corresponding biospecimen and clinical datasets were also retrieved from the GDC and curated to retain valid records only. All valid records came from the GDC‐enriched The Cancer Genome Atlas – Breast Invasive Carcinoma (TCGA‐BRCA) project. B) These datasets were subsequently preprocessed and used to build and evaluate a range of supervised learning models by tenfold CV with five repetitions. Because these datasets have high dimensionality, OMC models were also built to identify and retain the most important features.^[^ ^24^, ^53^ ^]^ 16 binary classification algorithms, 8 algorithms either with or without the OMC strategy, were applied to each of the 8 molecular profiles, resulting in the generated 128 models. Diverse binary classification metrics were used to evaluate the predictive performance of each developed model.

To our knowledge, ML is yet to be applied to predict BC patient response to doxorubicin treatments. Here we will evaluate a range of ML algorithms, which generally results in much better prediction than restricting to a single algorithm.^[^ ^24^, ^53^ ^]^ This is particularly true when considering algorithms that incorporate some form of feature selection to mitigate the impact of high‐dimensionality in the training data. The latter may have the additional advantage of providing interpretable molecular hypotheses for interpatient doxorubicin response variability. Integrating optimal model complexity (OMC) strategies with ML algorithms has resulted in improved predictions with problems spanning a range of drugs and cancer types.^[^ ^24^, ^53^, ^55^ ^]^ Another novel aspect of this study is that we will also investigate for the first time multivariate predictors based on patient response and profiles other than mRNA expression for this drug. The US National Cancer Institute (NCI) Genomic Data Commons (GDC) datasets have been harmonized across different cancer genome programs,^[^ ^56^ ^]^ providing a range of clinical drug response data and omics profiles. Thus, the data used in this study were obtained from the GDC repository (https://portal.gdc.cancer.gov). Beyond single‐gene and/or multigene expression predictors with modest performance,^[^ ^15^, ^23^ ^]^ we considered data from multiple molecular profiles arising from the application of various next‐generation sequencing technologies to patient samples and processed via various GDC workflows with their annotated treatment and biospecimen information. Drug response data of BC patients required the most intensive curation (**Figure** **1**A), as detailed in the Experimental Section. From a total of 64 drugs administered across BC patients, we could identify 96 of these patients (Table S1, Supporting Information) with both molecular profiling of their tumors and annotated responses to a doxorubicin‐containing treatment. Figure 1B presents the scheme of our methodology.

## 2 Results

Using data from the 96 BC patients (Table S1, Supporting Information), we employed 16 binary classification algorithms to generate and evaluate ML models to predict the responses to doxorubicin of these patients from the molecular profiles of their tumors. Figure 1 summarizes this process, which is fully specified in the Experimental Section. Figure S1 of the Supporting Information shows the high dimensionality of each profile, ranging from the expression of 927 miRNA isoforms (isomiR) to the methylation levels of 450 000 DNA probes.

### 2.1 Identifying the Most Predictive ML Algorithms and Molecular Profiles for Doxorubicin‐Response Prediction in BC Patients

**Figure 2:**
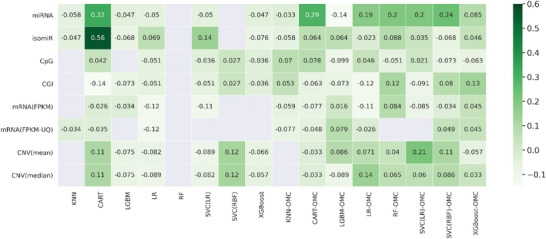
Heatmap showing the median MCC (mMCC) of five tenfold CV runs for each of the 128 models. Each row corresponds to a given molecular profile, each column refers to the employed classification algorithm. The first 8 algorithms on the left were ran without OMC and hence each lead to an all‐features model considering all available features from the processed datasets during model building. The rest of algorithms (the other 8 on the right) were ran with OMC to search for a small subset of features facilitating the classification of patients, thus the suffix “OMC” was added to the algorithm name. 10‐by‐10 nested‐CV runs were carried out five times, each time with a different random seed. For each run, CV predictions were merged to calculate the evaluation metrics. In this way, five MCC scores are obtained for each algorithm and molecular profile binomial, with its mMCC being shown in the heatmap. The two most predictive models were built with decision trees: mMCC of 0.56 from CART using isomiR features and mMCC of 0.32 from CART using miRNA features. However, the general trend is the OMC model (right) having higher MCC than its corresponding all‐features model (left). Models resulting in undefined mMCC scores are indicated as blank grey boxes. Here MCC is undefined because the model predicts the same class for all instances (e.g., if all are predicted positive, then the sum of true negatives and false negatives is by definition zero, which in turn makes the MCC denominator zero as well).

The accuracy of each set of tenfold CV (10CV) predictions for the patients by each model is quantified by its MCC. The median MCC (mMCC) of five 10CV repetitions, each with a different initial random seed, is presented in **Figure** **2**. Two models were able to distinguish between responders and nonresponders with an mMCC of at least 0.3, including mMCC of 0.56 from CART using isomiR features and mMCC of 0.32 from CART using miRNA features. Note that most models obtain a near‐random predictive level (MCC ≈ 0), with perfect prediction (MCC = 1) being still far away from the best models (this was expected, as we will discuss later). Figure S2 of the Supporting Information presents the results using other evaluation metrics, which evidences that the more common AUC is a less demanding metric than MCC. Indeed, AUC tends to overestimate predictive performance in imbalanced classification problems,^[^ ^57^, ^58^ ^]^ which is not the case of MCC.^[^ ^59^ ^]^ This is our main reason to use MCC as the primary performance metric.

Looking at the seven models with an mMCC of at least 0.2 (Figure S3, Supporting Information), all were built with algorithms inducing feature selection. Two‐thirds were OMC models, whereas the rest were all‐features CART models. Six of these models employed either isomiR or miRNA features. An inspection of the best results in terms of median AUC (mAUC) and their corresponding mMCC (Figure S4, Supporting Information) shows that these models also have high mAUC (CART_isomiR with an mAUC of 0.80 and CART_miRNA with an mAUC of 0.64). Noticeably, isomiR gives a better prediction of nonresponders when compared to miRNA (Table S2, Supporting Information). By contrast, Figure S4 of the Supporting Information also reveals many models with good mAUC but poor mMCC such as miRNA_LGBM (Light Gradient Boosting Machine). The latter model has 14 false negatives and 9 false positives, whereas CART_isomiR only has 4 false negatives and 3 false positives (Table S2, Supporting Information). This further supports the use of MCC for this type of class‐imbalanced problems.

### 2.2 Determining the Robustness of the Best ML Models

**Figure 3:**
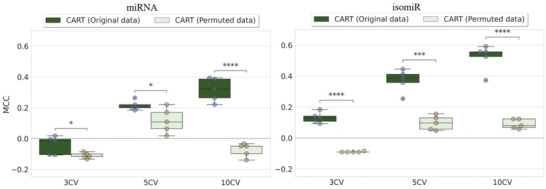
CV performance of the two most predictive models compared to those from training with permuted data. The boxplots present the distributions of MCC scores obtained across five iterations of CART models evaluation implemented on miRNA (left) and isomiR (right) datasets with 3CV, 5CV, and 10CV. CART models trained on the original dataset, i.e., all‐features data (deep green), and CART model trained on the class permuted dataset (light green). Each model's predictive performances (original and permuted) are compared within each of the CVs implemented for isomiR and miRNA. The horizontal bars above the boxplots indicate the significance levels between these distributions: “*” means 0.01 < *p* < = 0.05, “**” means 0.001 < *p* < = 0.01, “***” means 0.0001 < *p* < = 0.001, and “****” means *p* < = 1.00 1.00 × 10^−4^. These *p*‐values were calculated using two‐sided Welch's *t*‐tests. CART models obtain significantly better MCC than the permuted models in all the CVs, both with miRNA and isomiR features. Each dot represents a repetition.

After identifying isomiR_CART and miRNA_CART as the best ML models, here we evaluate how their predictive accuracies vary with different training set sizes and random seeds. With this purpose, we also conducted five runs of threefold CV (3CV) and fivefold CV (5CV) experiments, in addition to the five 10CV runs from the previous section, using different random seeds. Both models were robust to this type of variability given the similar MCC values returned within each set of experiments summarized by a boxplot (**Figure** **3**). Higher MCC values were observed as larger training sets were employed (from training with 67% of the data in 3CV to training with 90% of the data in 10CV). To find out which part of the predictive accuracy comes from signal in the data, we repeated all the CV runs exactly in the same manner from class‐permuted versions of the datasets. As a result, all the latter runs obtained near random‐level MCC values, which were significantly worse than those arising from models trained on the original data in all cases (Figure 3).

#### 2.2.1 Impacts of Data Integration on Doxorubicin Response Prediction

**Figure 4:**
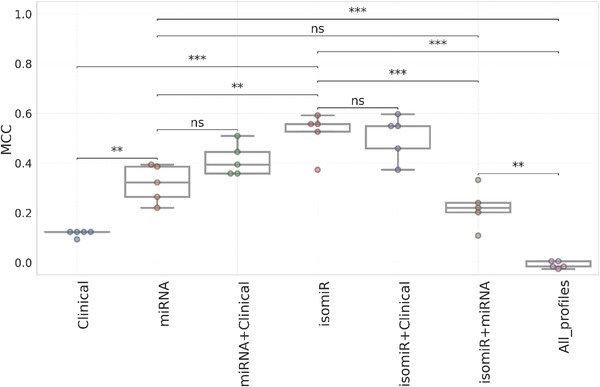
Comparison of the predictive performances of the model trained on the predictive molecular profile to those combining other datasets: Boxplots comparing the MCCs obtained from five runs of tenfold CV for the most predictive profiles (miRNA and isomiR) with those combining clinical data, merged both profiles (isomiR + miRNA) and those merging all the profiles considered in this study (clinical + 8 molecular profiles). The predictive models combining clinical and demographic information (Table S1, Supporting Information) with each of miRNA and isomiR profiles slightly performed better than those trained on miRNA and isomiR individually, but the differences were not statistically significant (*p* > 0.05). However, when the two predictive profiles were combined (miRNA + isomiR), a significant decrease in performance was observed compared with individual predictive profiles. Finally, the model combining all the 8 molecular profiles with clinical data has the least predictive performances; this could be due to the curse of dimensionality. It was observed that the higher the dimension of our dataset, the lower the performance of the models on the molecular profiles. Statistical comparisons between different models were performed using Welch's *t*‐test (two‐sided). Stars denote *p*‐value of the test, where; nonsignificant “ns” means 0.05 < *p* < = 1.00, “*” means 0.01 < *p* < = 0.05, “**” means 0.001 < *p* < = 0.01, and “***” means 0.0001 < *p* < = 0.001. Each dot represents a repeat.

We next look at whether adding or considering other sets features to describe patients improves prediction further. CART using clinical data was barely predictive (**Figure** **4**; Table S3, Supporting Information). These clinical features comprised sex, age, tumor stage, histological type, menopause status and the status of the estrogen, progesterone, and HER2 receptors (Table S1, Supporting Information). CART with merged clinical and isomiR features did not result in an improvement in MCC. However, integrating clinical and miRNA data improved MCC, although the difference with using miRNA features alone was not significant. We also evaluated CART with merged isomiR and miRNA features, which led to worse MCC values than using either profile alone. Lastly, CART using all the profiles as features led to the worse MCC overall.

#### 2.2.2 Comparing the ML Models to the Existing Single‐Gene Marker of Doxorubicin Response

**Figure 5:**
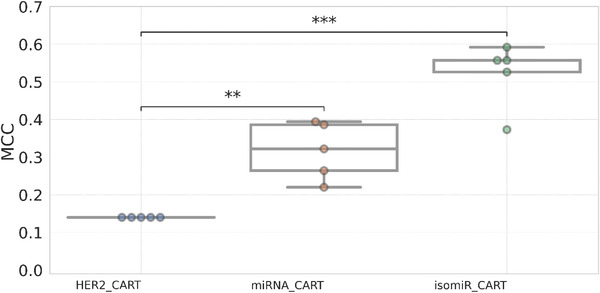
Comparison of the HER2‐based model with the best ML models to predict doxorubicin response in BC patients. Boxplots comparing the MCCs of CART models using miRNAs and their isoforms with those obtained using HER2 expression values only. HER2 expression (left) gave a significantly lower (*p* < 0.01) predictive performance than the two best ML models from this study (right). Legend: “**” means 0.001< *p* < = 0.01 and “***” means 0.0001 < *p* < = 0.001. The *p‐*values were calculated using two‐sided Welch's *t*‐test. Each dot represents a repeated run.

Gennari et al.,^[^ ^60^ ^]^ Rody et al.,^[^ ^22^ ^]^ Zhang and Liu^[^ ^61^ ^]^ showed independently that HER2 status is a marker of doxorubicin sensitivity in BC, with higher HER2 expression indicating higher sensitivity to the drug. **Figure** **5** shows that the mMCC obtained from the HER2 model is just 0.14, which is significantly lower than our predictive models with 3 miRNAs (mMCC = 0.32) or with 4 isomiRs (mMCC = 0.56). Table S4 of the Supporting Information also displays a sharp difference in terms of AUC. These results suggest that these ML models are able to predict doxorubicin response much better than the HER2 marker.

### 2.3 Assessing the Applicability Domain of the Best ML Models

**Figure 6:**
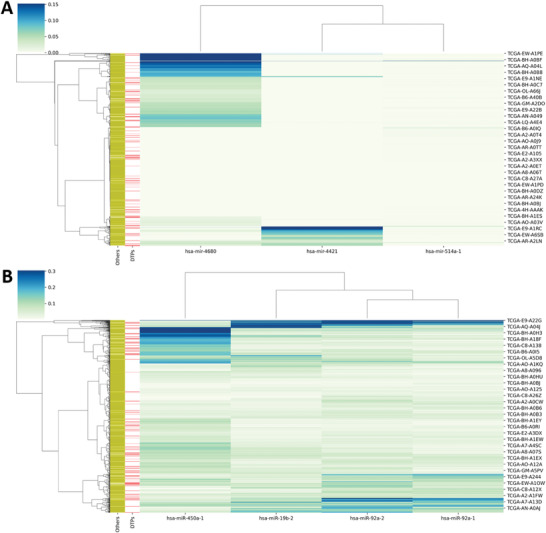
Expression of the selected miRNAs and isomiRs in 1078 TCGA‐BRCA cases. Clustering on the RPM‐normalized expression for the A) 3 selected miRNAs and B) 4 selected isomiRs with all the 1078 TCGA‐BRCA patients having both miRNA and isomiR data (with and without treatments records). The dendrogram on the left shows the clustering of patients, while the one on the top shows the clustering of the selected features (3 miRNAs and 4 isomiRs). The colors on the top‐left of the heat map represent the scale of the normalized expression values, while the colors in the heat map represent the expression intensities of the selected subsets of features. For the selected miRNAs (A), hsa‐miR‐4680 has more cases with the highest expression values among the 3 selected across all the cases considered, has‐miR‐4421 followed this and hsa‐miR‐514a‐1 has the least expression values as indicated by color partitions in the heatmap. Similarly, the column dendrogram showed that the expression of has‐miR‐4421 and hsa‐miR‐514a‐1 are more similar (i.e., both were in the same cluster) while that of hsa‐miR‐4680 are less similar to the other 2 miRNAs as indicated by the distance observed between the clusters. For the selected isomiRs (B), hsa‐miR‐450a‐1 has more cases with higher expression values when compared with others, while the remaining isomiRs (hsa‐miR‐19b‐1, hsa‐miR‐92a‐2, and hsa‐miR‐92a‐1) have very similar expressions across all the cases. Despite their similarity, hsa‐miR‐19b‐2 still has slight dissimilarity from other 2 and as such, it belongs to another cluster, whereas hsa‐miR‐92a‐2 and hsa‐miR‐92a‐1 form a cluster. Patients belonging to the same cluster/group are more similar and are less similar to patients in other clusters. For readability, the labels to the right of the *y*‐axis only present 36 patient IDs from different clusters out of 1078 included in each plot (this was done by minimizing the figure size). On the left of the dendrogram, the DTPs distribution is shown in red against other cohorts in yellow. There is no clear separation between the DTPs and others cohorts in both plots, which shows that the 95 patients are representative of the full 1078‐patient cohort.

We are not aware of publicly‐available BC datasets with other miRNA‐profiled doxorubicin‐treated patients. However, there is a total of 1078 miRNA‐profiled patients in the GDC TCGA‐BRCA project. Therefore, we can investigate whether the subset of 95 miRNA‐profiled doxorubicin‐treated patients constitutes a representative sample of all these patients. **Figure** **6**A shows how these 1078 patients cluster by their similarity in the expression of predictive miRNA features. (Figure 6B shows the clustering of the same patients with respect to predictive isomiR features.) In both cases, the 95 doxorubicin‐treated patients are evenly distributed across clusters, which means that all major clusters are represented in the training set of each of the best ML models we identified. This lack of a strong distribution shift between the training and potential test sets suggests that these models should also be predictive on the rest of miRNA‐profiled patients.

### 2.4 The Best ML Models Are Also Highly Interpretable

**Figure 7:**
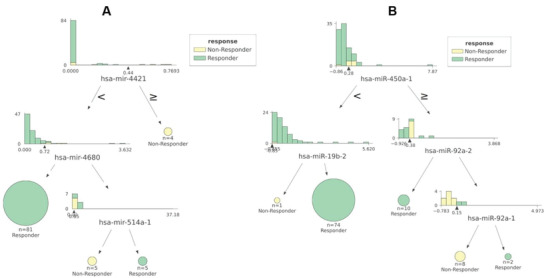
Interpreting the most predictive models based on miRNA and isomiR expression data. A) CART with the 3 selected miRNA features (hsa‐miR‐4421, hsa‐miR‐4680, and hsa‐miR‐514a‐1) trained on all 95 patients with miRNA profile. The histogram in each tree node shows the distribution of patients at that node against the feature employed to split the patients into nonresponders and responders. The triangle under each histogram indicates the value of the best split for that feature, whose name can be found beneath the histogram. Each node has two leaves: to the right (patients with a feature value greater than or equal to the best split) and the left (the rest of the patients). Terminal nodes appear as circles and the decision path to get to each of them constitute an explanation of why a patient has been assigned the associated response class. For instance, there are two molecular subtypes in miRNA space associated to doxorubicin resistance: i) patients whose tumors express hsa‐miR‐4421 ≥ 0.44 are predicted to be nonresponders (4 out of the 9 nonresponders have tumors verifying this decision rule in the trained model), and ii) patients whose tumors express hsa‐miR‐4421 < 0.44, hsa‐miR‐4680 ≥ 0.72 and hsa‐miR‐514a‐1 < 0.65 are also predicted to be nonresponders (the remaining 5 nonresponders follow this decision rule). Otherwise, the patient is predicted to be a responder. B) CART with the 4 selected isomiR features (hsa‐miR‐450a‐1, hsa‐miR‐19b‐2, hsa‐miR‐92a‐2, and hsa‐miR‐92a‐1) trained on all 95 patients. In the isomiR space, there are also two molecular subtypes associated to doxorubicin resistance: i) patients with tumors expressing hsa‐miR‐450a‐1 ≥ 0.28, hsa‐miR‐92a‐2 ≥ ‐0.38, and hsa‐miR‐92a‐1 < 0.15 are predicted to be nonresponders (8 out of the 9 nonresponders have tumors verifying this decision rule in the trained model), and ii) patients whose tumors express hsa‐miR‐450a‐1 < 0.28, and hsa‐miR‐19b‐2 < ‐0.85 are also predicted to be nonresponders (the remaining nonresponder follow this decision rule). Conversely, the patient is predicted responder.

Out of the 16 models evaluating miRNA features, CART was found to be the most predictive. This model selected 3 (hsa‐miR‐4421, hsa‐miR‐4680, and hsa‐miR‐514a‐1) out of the 1881 considered miRNAs (**Figure** **7**A; Table S2, Supporting Information). On the other hand, among the models employing isomiR features, only CART was substantially predictive by selecting 4 (hsa‐miR‐450a‐1, hsa‐miR‐19b‐2, hsa‐miR‐92a‐2, and hsa‐miR‐92a‐1) out of 927 analyzed isomiR features (Figure 7B; Table S2, Supporting Information).

#### 2.4.1 Predicted miRNA Target Genes

A miRNA is a small single‐stranded noncoding RNA molecule able to bind to a specific set of mRNA molecules. Such binding results in either degrading the bound mRNA molecule or suppressing its translation into a protein.^[^ ^62^ ^]^ That is, an miRNA influences the post‐transcriptional regulation of a specific set of protein‐coding genes (its targeted genes). A total of 13898 genes are predicted to be targeted by the 7 predictive miRNAs (**Table** **1**) according to TargetScan.^[^ ^63^ ^]^

**Table 1:** 23 BC‐driving genes are likely to be targeted by the 7 predictive miRNAs

miRNA	TargetScan	TargetScan ∩ IntOGen	TargetScan ∩ COSMIC	TargetScan ∩ COSMIC ∩ IntOGen
hsa‐mir‐4421	3284	22	11	7
hsa‐miR‐4680‐3p	4870	43	16	9
hsa‐miR‐4680‐5p	3864	24	5	4
hsa‐miR‐514a‐3p	3520	28	9	7
hsa‐miR‐514a‐5p	3851	31	12	10
hsa‐miR‐450a‐1‐3p	6030	34	14	9
hsa‐miR‐19‐5p	4906	37	12	8
hsa‐miR‐92a‐2‐5p	5873	36	16	10
hsa‐miR‐92a‐1‐5p	3287	26	7	4
# of unique genes	13 898	89	37	23

**Figure 8:**
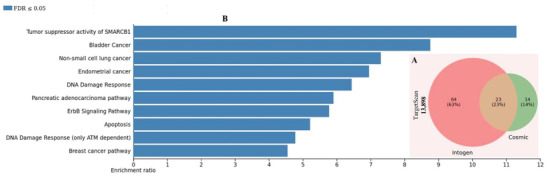
A) Venn diagram presenting the number and percentage of genes TargetScan‐mapped to be targeted by the 7 predictive miRNAs in IntOGen and COSMIC databases (23 of these genes overlapped between the three gene lists). B) EA of the 23 overlapping target genes using WikiPathway cancer. The bar chart summarizes 10 biological pathways that are significantly (FDR < 0.05) enriched with different subsets of the 23 selected target genes for the predictive miRNAs. The bars represent the enrichment ratio (ER, as the number of gene overlaps over its expected value) obtained by comparing the gene list in our study with the reference gene set from genome protein‐coding. FDA: false discovery rate.

To unveil BC‐associated processes, we first determined the overlap between TargetScan genes and the 89 and 37 genes that are reported to be BC‐driving genes in Integrative OncoGenomics (IntOGen)^[^ ^64^ ^]^ and Catalogue of Somatic Mutation in Cancer (COSMIC)^[^ ^65^, ^66^ ^]^ databases, respectively. To enhance the BC‐specificity of the analysis, we focus on the 23 genes that we found to be common to these three gene lists (**Figure** **8**A): AKT1, ARID1A, ARID1B, BAP1, BRCA1, BRCA2, CASP8, CDH1, CDKN1B, CTCF, ERBB2, ESR1, FOXA1, GATA3, MAP2K4, MAP3K1, NCOR1, PIK3CA, RB1, SALL4, SMARCD1, TBX3, TP53 (Table S6, Supporting Information).

#### 2.4.2 Enrichment Analysis (EA) of the Genes Targeted by the Predictive miRNAs

We next carried out EA using the over representation analysis (ORA) method implemented in the WEB‐based Gene SeT AnaLysis Toolkit (WebGestalt).^[^ ^67^ ^]^ ORA was used to identify the biological pathways significantly enriched with the 13898 targeted genes from the cancer‐related repository of WikiPathways (WikiPathway cancer). The four cancer‐associated pathways that were found (false discovery rate (FDR) ≤ 0.05) are DNA damage response, ErbB (epidermal growth factor receptor family) signaling pathway, endometrial cancer, and chromosomal and microsatellite instability in colorectal cancer (Table S5, Supporting Information). Figure S5A of the Supporting Information complements this information with the gene ontology (GO) terms, including biological process, cellular component, and molecular function, that are enriched with these 13898 genes. Pathways associated with doxorubicin's mechanism of action such as DNA damage response^[^ ^68^, ^69^ ^]^ and cell signaling,^[^ ^70^ ^]^ were found to be enriched. This suggests that dysregulation of the predicted target genes involved in these pathways could promote doxorubicin resistance. Also, alterations in ErbB signaling pathways could exacerbate breast tumorigenesis,^[^ ^71^ ^]^ which would contribute to doxorucibin seeming less effective, and have been linked to doxorubicin resistance.^[^ ^72^ ^]^

Aiming at revealing the most predominant BC‐specific biological processes underpinning primary resistance to doxorubicin, EA was conducted for the 23 genes obtained from leveraging BC‐driver knowledge consensus and miRNA target prediction (Table 1). ORA was now used to identify biological pathways enriched with these 23 genes from both repositories WikiPathway cancer and Kyoto Encyclopedia of Genes and Genomes (KEGG). This gene list was significantly (FDR ≤ 0.05) enriched in 10 cancer‐associated biological pathways each, as indicated in both WikiPathway cancer (Figure 8B; Table S6, Supporting Information) and KEGG (Figure S5C, Supporting Information), three of these pathways (breast cancer, pancreatic cancer, and endometrial cancer) are common to both WikiPathway cancer and KEGG and appeared enriched with the same sets of genes. Other pathways also emerged significantly enriched (FDR ≤ 0.05) in WikiPathway of cancer, including tumor suppressor activity, DNA damage response, apoptosis, cell signaling pathway, bladder cancer, and nonsmall cell lung cancer pathway. Of note, 8 of these 23 genes (ERBB2, ESR1, AKT1, PIK3CA, RB1, TP53, BRCA2, BRCA1) specifically overlapped with the BC annotated gene set (Figure S6 and Table S6, Supporting Information) in both WikiPathway cancer and KEGG. The GO terms enriched with these 23 genes are presented in Figure S5B of the Supporting Information. Due to doxorubicin‐mediated DNA damage, apoptotic cell death can be induced via the activation of tumor suppressors in the cell cycle control and apoptosis.^[^ ^73^ ^]^ However, defect in these regulators can lead to doxorubicin failing to induce cell cycle arrest and apoptosis.^[^ ^68^, ^69^ ^]^ The 23 BC‐specific genes regulate processes associated with doxorubicin's mechanism of action and the breast cancer pathways in WikiPathway cancer (Figure 8). These genes are also part of drug resistance KEGG pathways, such as second‐ranked platinum drug resistance and third‐ranked endocrine resistance pathways (Figure S5C, Supporting Information), which are not found in enrichment analysis of the 13898 genes (Table S5, Supporting Information).

## 3 Discussion and Conclusions

Many studies^[^ ^15^, ^74^ ^]^ have focused on identifying mutation‐based single‐gene markers and/or gene expression signatures for precision oncology. With fast‐growing clinical pharmaco‐omic datasets being available in the public domain, ML has become a highly promising approach to discover how these molecular factors could collectively explain and predict drug response.^[^ ^14^, ^53^, ^54^, ^75^ ^]^

Owing to the wealth of curated profiling data from the GDC, we could carry out an unusually broad analysis covering eight tumor profiles per patient. In line with previous studies analyzing other drugs,^[^ ^52^, ^53^ ^]^ this large‐scale analysis across multiple profiles and algorithms has resulted in the identification of the first ML models able to predict patient response to doxorubicin‐containing treatments (CART allied with either miRNAs or their isoforms). Owing to CART's embedded feature selection, these CART models only employ either three or four features of the about 1–2 thousand considered. Another sign of the importance of feature selection when training on high‐dimensional datasets is that OMC models were generally more predictive than their all‐features counterparts. Overall, only 2 of the 128 models in this large‐scale analysis were substantially predictive (Figure 2), showing that they can be easily missed by a standard‐scale analysis. Interestingly, the fact that the best models are nonlinear, despite testing linear models too, hints the nonlinearity of the data.

Merging features of potentially complementary nature yielded mixed results (Figure 4). While merging clinical and miRNA features improved the accuracy of miRNA features alone, the difference was not significant and this improvement was not observed with isomiRs. Furthermore, clinical features alone were barely predictive and combining them with all the molecular profiles led to an almost complete loss of predictive accuracy. We attribute the latter to CART not being able to cope with the far higher number of features involved (over half a million). In fact, higher dimensionality is detrimental well before 3000 features (i.e., merging miRNAs and their isoforms, as this results in CART models with lower MCC values than any of the two profiles in isolation). Thus, as no improvement is achieved by combining the most predictive profiles, determining multiple profiles per tumor is not recommended due to also being much more expensive and time‐consuming.

The predictive accuracy of the isomiR‐based CART model is high in the context of this problem despite the strong class imbalance. First, the mMCC of its tenfold CV predictions across five independent repetitions is 0.56, which corresponds to a mAUC of 0.80. Importantly, while all models with a substantial mMCC also have a substantial mAUC, the opposite is not true. For instance, the miRNA‐based LGBM model returned an mAUC of 0.71, but its mMCC is practically zero. In class‐imbalanced problems like this, we recommend MCC as a more appropriate alternative to AUC, as the latter has here overestimated the ability of some models to discriminate between responders and nonresponders. Second, the best ML models are much more predictive than their respective permuted versions (Figure 3). Third, HER2 expression was identified as a doxorubicin response marker, as also found by other studies supporting the use of this single‐gene marker,^[^ ^22^, ^60^, ^61^ ^]^ but its mMCC is just 0.14 (Figure 5), which is four times lower than the mMCC of the best ML model. Lastly, our best predictive model with mMCC of 0.56 compares well to the very few existing in vivo treatment response ML models for other drugs and cancer types (MCC ranging from 0.36 to 0.54^[^ ^24^, ^76^, ^77^ ^]^).

To go beyond these retrospective validations, we performed clustering on an additional 1078 BC patients profiled for both miRNAs and isomiRs (Figure 6). Here we showed that, in either of these profiles, the 95 doxorubicin‐treated patients in the training set were well represented in all the clusters in which the 1078 BC patients are partitioned. This means that these patients are within the applicability domain of these models, which are therefore expected to have similarly high accuracy when predicting their response to doxorubicin. Furthermore, the predictive accuracies of these models were robust to using different data partitions and algorithm initializations (Figure 3). This further supports that similarly small fluctuations in accuracy should be observed on other BC patients.

An additional advantage of the identified models is that they are interpretable at the patient level. For example, a patient is predicted to be a nonresponder because her/his expression levels for the 3 selected miRNAs, hsa‐miR‐4421, hsa‐miR‐4680, and hsa‐miR‐514a‐1, are nonlinearly combined in a way that has been accurately associated to nonresponders by the CART algorithm (Figure 7A). This 3‐miRNA model is further supported by the relevant individual roles of each of their constituting miRNAs. For instance, hsa‐miR‐4421 and hsa‐miR‐4680 are overexpressed in luminal A BC^[^ ^78^ ^]^ and hereditary BRCA2 BC,^[^ ^79^ ^]^ respectively. Also, hsa‐miR‐514a‐1 negatively correlates with BC recurrence.^[^ ^80^ ^]^ On the other hand, the second CART model presents predictive combinations of 4 isomiRs: hsa‐miR‐450a‐1, hsa‐miR‐19b‐2, hsa‐miR‐92a‐2, and hsa‐miR‐92a‐1. This model is also supported by the relevant individual roles of these four molecules. hsa‐miR‐450a‐1 is upregulated in BC.^[^ ^81^ ^]^ Also, overexpression of hsa‐miR‐19b family members are candidate prognostic biomarkers of BC and their involvement in the tumor progression through the PI3K/AKT pathway has also been documented.^[^ ^82^, ^83^ ^]^ Downregulation of hsa‐miR‐92a family members is also associated with aggressive BC and high tumor macrophage infiltration.^[^ ^84^ ^]^ Hsa‐miR‐92a family members are also involved in the formation of blood vessels and the development of some mammalian organs,^[^ ^85^ ^]^ with their aberrant expressions being highly associated with different malignant human tumors.^[^ ^86^, ^87^, ^88^ ^]^ Therefore, they have been reported as a potential therapeutic target and novel diagnostic biomarker of human tumors.^[^ ^89^ ^]^ In addition, hsa‐miR‐92a is involved in apoptosis, cell proliferation, and doxorubicin chemosensitivity in gastric carcinoma cells, with the suppression of hsa‐miR‐92a leading to DNA damage foci and thus sensitivity to doxorubicin treatment.^[^ ^90^ ^]^

In addition to doxorubicin response, individual miRNAs are often associated to other cancer patient outcomes. They play significant roles in RNA silencing as well as post‐transcriptional regulation of gene expression.^[^ ^91^, ^92^ ^]^ As miRNAs play critical roles in gene regulation and drug resistance, their dysregulation could promote cancer development, recurrence, and chemoresistance.^[^ ^93^, ^94^ ^]^ MiRNAs can be used as tools or targets for the treatment of different cancers^[^ ^95^ ^]^ because of their essential roles as gene regulators for various human cancers.^[^ ^96^ ^]^ Hundreds of genes, including oncogenes and tumor suppressor genes, can be regulated by a single miRNA binding to their mRNA transcripts.^[^ ^97^, ^98^ ^]^ Conversely, a single mRNA transcript can bind different miRNAs. That is, one single miRNA usually targets many genes and different miRNAs might regulate the same gene.^[^ ^91^ ^]^

To reveal the most predominant BC‐specific biological processes underpinning primary resistance to doxorubicin, EA for the 23 predicted BC‐associated genes targeted by our predicted miRNAs revealed ten significantly (FDR < 0.05) enriched cancer‐associated biological pathways (Figure 8B), including tumor suppressor activity, apoptosis, DNA damage response, bladder cancer, nonsmall cell lung cancer, endometrial cancer, BC pathway among others (Table S6, Supporting Information). Eight of these genes were enriched in the BC pathway (Figure S6, Supporting Information), five in DNA damage response, five in apoptosis, and so on (Table S6, Supporting Information). Details of the enriched genes targeted by each miRNA are reported in Table S8 of the Supporting Information. Deregulation of any of the components of the identified pathways listed above leads to aberrant expression of several associated genes, consequently resulting in different disorders or diseases, including cancer.^[^ ^99^ ^]^ For example, the deregulation of our predicted target genes in DNA damage and apoptotic pathways could be a mechanism that promotes patients resistance to doxorubicin, whose mechanism of action include inducing DNA damage to trigger apoptotic cell death.^[^ ^7^ ^]^ This EA provides starting points for studies investigating the molecular mechanisms of primary resistance to doxorubicin in BC patients.

Our study has some limitations to point out. While clustering analysis has shown that the best ML models are likely predictive on an additional cohort with over a thousand BC patients whose tumors were profiled for miRNAs and isomiRs, this is still to be confirmed prospectively. In such prospective clinical trial, each BC patient would only have to be profiled pretreatment for the 4 isomiRs selected by CART, which would represent a large saving in time and cost with respect to determining the full profile. Another limitation is that the TCGA BRCA project collected treatment response data on a voluntary basis from hundreds of submitting institutions. Thus, the level of curation and harmonization of these datasets is likely to be much lower than that of the molecular profiles. With that said, there is a strong signal in treatment response data, at least for the 96 patients used for training, given that models trained on permuted response data were significantly less predictive.

Overall, this large‐scale ML analysis has led to the discovery of highly predictive, robust and even interpretable predictors of BC patient response to doxorubicin. These are CART models nonlinearly combining selected miRNAs and their isoforms in a predictive manner (EA of the genes potentially regulated by these molecules provide starting points for mechanistic studies). These CART models achieved median MCC values that are at least four times higher than those based on HER2 expression and response‐permuted data. Importantly, the MCC of these models increased with larger training sets, therefore these should become even more predictive as more data are available in the future.

## 4 Experimental Section

### Acquisition, Retrieval, and Preprocessing of Drug Response Data

The open‐access clinical and biospecimen files of the primary tumor samples in the TCGA‐BRCA project were downloaded from the GDC portal (version 25.0, July 22nd, 2020 release). Note that this TCGA acronym stands for Breast Invasive Carcinoma, not the BRCA gene. To avoid confusion, BC was employed to refer to this cancer type, for which TCGA contains data for 1098 patients. To curate these datasets, misspellings, formulations, and synonyms (e.g., adriamycin, doxil, doxorubicinum, liposomal doxorubicin, and doxorubicin liposome) of the drug as annotated in the DrugBank database^[^ ^100^ ^]^ to its generic name (doxorubicin), were first standardized. Next, the 332 BC patients who received doxorubicin were identified. Among these patients, those without annotated doxorubicin responses, those missing sample collection and treatment start dates were excluded. Thus, 236 (71.1%) of patients were excluded from the study for these reasons. After these filtering steps, 96 BC patients treated with doxorubicin were retained (these patients did not receive chemotherapy prior to tumor sampling, as indicated by the times of tumor sample procurement and the start of treatment). 78 (81.3%) of whom had a tumor biopsy taken, while the remaining 18 (18.7%) had their tumors surgically resected instead. Patient responses to doxorubicin‐containing treatments, complete response (CR), partial response (PR), stable disease (SD), and progressive disease (PD), were provided by the TCGA‐BRCA project. As it is common practice,^[^ ^26^, ^101^ ^]^ such responses were further categorized into two classes: responder (CR or PR) and nonresponder (SD or PD). This process resulted in a total of 87 responders and 9 nonresponders (Table S1, Supporting Information).

### Acquisition, Retrieval, and Preprocessing of Molecular Profiling Data

The open‐access molecular profiles for the 1098 TCGA‐BRCA patients were also downloaded from the GDC portal. This was restricted to baseline primary tumor samples (i.e., those annotated with “01” in the 14th and 15th positions of the TCGA sample code). A few patients had multiple tumor samples sequenced. In this case, the first labeled sample was chosen, which corresponds to the A‐level sample (e.g., TCGA‐02‐0001‐01A). To reduce technical variability, the primary analysis of profiling data was harmonized by the GDC (i.e., carried out according to the same standardized GDC workflows). All the nonrestricted‐access profiles were considered for these patients. Thus, eight molecular profiles per patient were considered: mRNA(FPKM) are the messenger RNAs as Fragment Per Kilobase of transcript per Million, mRNA(FPKM‐UQ) are the messenger RNAs as Upper‐Quantile‐normalized FPKM, miRNA are the log2‐transformed Reads Per Million mapped‐normalized microRNAs, isomiR are the log2‐transformed Reads Per Million mapped‐normalized miRNA isoforms expression at a given locus that are distinguished by their location within the locus, CpG are the DNA methylation beta values of 450k probe at known CpG sites, CGI are the averaged beta values of all the probes at the CpG sites of the corresponding CpG Island, CNV(mean) are the Copy Number Variants calculated using CNTools R packages as the mean of DNA copy number across the segments of the considered gene, and CNV(median) is calculated in the same as CNV(mean) except using the median instead of the mean. Each molecular profiling dataset was used as a set of features for building models using a range of ML algorithms.

### Preparing Datasets for ML

Only some doxorubicin‐treated BC patients have both treatment responses and molecularly profiles annotated. The number of doxorubicin treated BC patients along with the number of features of each dataset was reported in Figure S1 of the Supporting Information. Each individual dataset was split into training and testing set using stratified K‐fold^[^ ^102^ ^]^ CV, with K values being 3, 5, and 10. The predictions from each left‐out CV fold were merged prior to calculating a given evaluation metric (e.g., MCC), instead of calculating the metric for each left‐out fold and average them. This provides a more robust estimation of the metric, while ensuring that the prediction of each instance from the CV was not used in any way for the training or selection of its corresponding model.

The median results from the 5 seeds are reported for each of the 128 ML models in Figure 1 (16 ML models × 8 molecular profiles). Because of the class imbalance of the processed datasets, the class weights were applied inversely proportional to class frequencies during model fitting. The process was repeated five times with different random seeds. The median of performance metrics of five repetitions was reported. All analyses were performed using python package version 3.7.3 (https://www.python.org/) with packages from scikit‐learn (https://scikit‐learn.org).

### Building All‐Features Classification Models

8 ML algorithms were employed: CART,^[^ ^103^ ^]^ random forest,^[^ ^104^ ^]^ XGBoost,^[^ ^105^ ^]^ LGBM,^[^ ^106^ ^]^ Logistic regression,^[^ ^107^ ^]^ Linear Support Vector Machine and Radius Support Vector Machine,^[^ ^108^ ^]^ and K‐Nearest Neighbors.^[^ ^109^ ^]^ The model hyperparameters were set to their default values.

Standard stratified K‐fold CVs were performed to measure the model performance for each algorithm‐profile pair using all the available features for that profile. This is called an all‐features model (however, as some algorithms such as CART possess embedded feature selection, the resulting model will only employ a fraction of the features to calculate its predictions). During CV, K‐1 folds were used for model training, whereas the remaining partition was used to test the trained model. Thus, each fold was used exactly once as a test set. Therefore, for any given model, the response of a patient was predicted using a model trained with data from other patients.

### Building OMC Classification Models

Due to the high dimensionality of the datasets (i.e., the number of features is much larger than the number of patients), OMC^[^ ^24^, ^53^ ^]^ variants of each of the 8 ML algorithms were implemented to build models using only the most relevant features of the doxorubicin response. This can remove noise from the training data and improve the model performance. To select models with the OMC while estimating their performance, nested CV was carried out for each algorithm‐profile pair. In brief, OMC is comprised of three steps. First, features were ranked according to their relevance to doxorubicin response by increasing *p*‐values from Analysis of Variance (ANOVA). The *p*‐value, one per each feature, indicates the discriminative power to distinguish between responders and nonresponders, the informative features associated with small *p*‐values. Then, an ML model was trained with considered subset of features (the top 2 to *n*/2 subset of features, where *n* is the number of samples). Finally, the best‐performing model was selected among all *n*/2 trained model as the one with the highest MCC in the inner loop of the nested CV and evaluated that model performance in its outer loop.

### Comparing the Best ML Models to Permutation and HER2 Expression Models

As a baseline, CVs for a top model was also run with the same algorithm and features, but after randomly shuffling doxorubicin response labels across patients. This was called a permutation model.

On the other hand, HER2 status has been identified as a single‐gene marker for BC patient sensitivity to doxorubicin in several studies.^[^ ^22^, ^60^, ^61^ ^]^ To compare the accuracy of the ML models at this task with that of using HER2 only, an HER2‐CART model was built using standard tenfold CV with HER2(ERBB2) expression data derived from the mRNA(FPKM) profile.

### Model Performance Evaluation

For MCC, as it is customary, the operating threshold was set to 0.5. Patients with class probabilities above this threshold were predicted to be responders, otherwise they were predicted to be nonresponders. To estimate predictive performance, the true and predicted classes were compared. The numbers of true positive, true negative, false positive, and false negative instances were used to calculate performance metrics. The AUC of the ROC, AUC for short, was also calculated for comparison purposes.

### Clustering and Pathway Analysis of BC Patients from Selected miRNA Features

BC patients were clustered using the predictive miRNA features (either miRNAs or isomiRs) with an agglomerative clustering algorithm (Ward's linkage with Euclidean distance to calculate similarity between the clusters of patients). A dendrogram heatmap was used to visualize the results of this clustering analysis, marking the 95 patients with high‐quality response and profiling data. Both clustering and its visualization were carried out with clustermap in the seaborn package (version 0.11.2).

### Data Processing for EA

To reveal the biological pathways related to predictive miRNAs, the target genes of predictive miRNAs were predicted using the TargetScan database (version 7.0).^[^ ^63^ ^]^ This online miRNA target prediction tool identify mRNA by matching the desired miRNA seed region to the conserved complementary sites (sequences) of their mRNA targets.^[^ ^110^, ^111^, ^112^, ^113^ ^]^ It retrieves the highest number of target genes of any database and ranks the predicted targets of each miRNA based on these matching algorithms.^[^ ^63^ ^]^ Then, the list of BC‐driving genes was download from IntOGen,^[^ ^64^ ^]^ and COSMIC.^[^ ^66^ ^]^ Of those, the subset of genes in common of TargetScan, IntOGen, and COSMIC were considered as potential BC miRNA targets. They were used as input data of EA to explore the biological relevance of predictive miRNAs. WEB‐based Gene SeT AnaLysis Toolkit (WebGestalt)^[^ ^67^ ^]^ (www.webgestalt.org) was utilized for EA for WikiPathway cancer, KEGG, and GO database. WebGestalt uses a hypergeometric test for statistical significance, which further employed the Benjamin–Hochberg FDR multiple‐testing correction. The enriched pathways were identified based on FDR threshold which was set to 0.05. For each pathway, input genes that are part of the pathway are counted and enrichment ratio was also calculated.

### Statistical Analysis

Preprocessing of data: See subsections entitled “Acquisition, Retrieval, and Preprocessing of Drug Response Data”, “Acquisition, Retrieval, and Preprocessing of Molecular Profiling Data”, and “Preparing Datasets for ML” at the start this section.

Data presentation: Whenever relevant, Figures 2, 3, 4, 5, 6, 7, 8 present the distributions of performance metrics (e.g., Figure 3). On the other hand, Table S1 of the Supporting Information presents all the clinical data.

Sample sizes for each statistical analysis: Each sample is formed by 5 runs whose results are summarized by boxplot (Figures 3, 4, 5). Each dot represents the model performance from that run (each using a different random seed). The provenance of each sample is specified in the figure captions.

Statistical methods: A two‐tailed Welch's *t*‐tests for each considered pair of samples. A performance difference was considered to be significant if *p*‐value < 0.05.

Software used for statistical analysis: Statistical analysis was carried out using the statannot package in the python 3.7.3 software.

Code availability: The python codes and processed datasets are provided to build the best classifiers and the HER2 baseline, evaluate them and facilitate their application to other cohorts of miRNA‐profiled BC patients: https://github.com/adeolu1/BRCA_CART_model.

## Conflict of Interest

The authors declare no conflict of interest.

## Author Contributions

P.J.B. conceived the idea and designed the experiments. A.Z.O. collected and analyzed the patients’ data, developed the ML process and interpreted the results. A.Z.O. and P.J.B. wrote the manuscript with the assistance of C. Piyawajanusorn, A. Gonçalves, and G. Ghislat. All authors contributed to the discussion.

## Supporting information

Supporting Information

## Data Availability

The data and the corresponding cancer information were downloaded from the Genomic Data Commons portal (https://portal.gdc.cancer.gov/) and were, in whole, based upon open access data generated from the TCGA‐BRCA project. Thus, these data were publicly available without restriction, authentication, or authorization.
