# Supplementary material for: Interpretable Machine Learning Models to Predict the Resistance of Breast Cancer Patients to Doxorubicin from Their microRNA Profiles
Source: Adv Sci (Weinh). 2022 Jul 3;9(24):2201501. doi: 10.1002/advs.202201501 (PMC9403644; doi:10.1002/advs.202201501)
Supplement: Supplementary file 1 — Supporting Information [file ADVS-9-2201501-s001.pdf]

SUPPLEMENTARY INFORMATION

Interpretable machine learning models to predict the resistance of breast cancer patients to doxorubicin from their microRNA profiles

Adeolu Z. Ogunleye[1], Chayanit Piyawajanusorn[1], Anthony Gonçalves[1], Ghita Ghislat[1] and Pedro J. Ballester[1*]

[1] Cancer Research Center of Marseille (CRCM), INSERM U1068, F-13009 Marseille, France; Institut Paoli-Calmettes, F-13009 Marseille, France; Aix-Marseille Université, F-13284 Marseille, France; and CNRS UMR7258, F-13009 Marseille, France.
[*]correspondence to: pedro.ballester@inserm.fr

Features	Possible values	All patients	Responder only	Non-responder only
Treatment response	Responder, non-responder	96	87 (90.62%)	9 (9.38%)
Sex	Female	95 (98.96%)	86 (90.53%)	9 (9.47%)
	Male	1 (1.04%)	1 (100%)	0 (0%)
Age (year)	Range	28 – 82	28 - 82	44 – 61
	Median ± SD	51 ± 10.2	51 ± 10.6	53 ± 5.6
Tumor Stage	Stage I & IA	14 (14.58%)	13 (92.86%)	1 (7.14%)
	Stage II (A & B)	49 (51.04%)	47 (95.92|%)	2 (4.08%)
	Stage III (A, B, & C)	32 (33.33%)	27 (84.38%)	5 (15.62%)
	Stage IV	1 (1.04%)	0 (0%)	1 (100%)
Race	White	47 (48.96%)	39 (82.98%)	8 (17.02%)
	Black or African American	33 (34.38%)	32 (96.97%)	1 (3.03%)
	Asian	10 (10.42%)	10 (100%)	0 (0%)
	Not reported	6 (6.25%)	6 (100%)	0 (0%)
Histological type	Ductal	60 (62.5%)	58 (96.7%)	2 (3.3)
	Lobular	28 (29.2%)	23 (82.1)	5 (17.9)
	Mixed ductal and lobular	6 (6.2%)	5 (83.3%)	1 (16.7%)
	Metaplastic	2 (2.1%)	1 (50%)	1 (50%)
IHC estrogen status	Positive	65 (67.7%)	57 (87.7%)	8 (12.3%)
	Negative	29 (30.2%)	28 (96.6%)	1 (3.4%)
	Not reported	2 (2.1%)	2 (100%)	0 (0%)
IHC progesterone status	Positive	54 (56.3%)	47 (87.0%)	7 (13%)
	Negative	40 (41.7%)	38 (95%)	2 (5%)
	Not reported	2 (2.1%)	2 (100%)	0 (0%)
IHC HER2 status	Positive	16 (16.7%)	15 (93.8%)	1 (6.2%)
	Negative	75 (78.1%)	68 (90.7%)	7 (9.3%)
	Equivocal	3 (3.1%)	2 (66.7%)	1 (33.3%)
	Not reported	2 (2.1%)	2 (100%)	0 (0%)
Menopause status	Post-menopause	56 (58.3%)	50 (89.3%)	6 (10.7%)
	Peri-menopause	7 (7.3%)	6 (85.7%)	1 (14.3%)
	Pre-menopause	22 (22.9%)	21 (95.5%)	1 (4.5%)
	Not reported	11 (11.5%)	10 (90.9%)	1 (9.1%)

HER2: Human epidermal growth factor receptor 2, IHC: Immunohistochemistry

Table S1: Patient cohort statistics. A description of the clinicopathological features of patients included in this study and their tumors (we also add treatment response for reference). For each patient, sex, age, menopause status, estrogen, progesterone and HER2 status, histological type, tumor stage, race and vital status, are reported. Eight of the 11 patients with HER2 equivocal status and 11 of the 13 patients with missing HER2 status were clarified based on FISH-HER2 status, while the remaining three and two cases with equivocal status and missing status respectively, have unreported data for both IHC and FISH. FISH: Fluorescence In Situ Hybridization.

**3 selected miRNAs**	**4 selected isomiRs**
hsa-miR-4680 (MIMAT0019764)	hg38:chrX:134540393-134540415:-(hsa-miR-450a-1/ MIMAT0001545)
hsa-miR-4421 (MIMAT0018934)	hg38:chrX:134169683-134169706:- (hsa-miR-19b-2 / MIMAT000007)
hsa-miR-514a-1 (MIMAT0002883)	hg38:chrX:134169544-134169566:- (hsa-miR-92a-2 / MIMAT0000092)
	hg38:chr13:91351361-91351382:+ (hsa-miR-92a-1 / MIMAT0000092)

Confusion Matrix: miRNA	
TP = 78	FP = 4
FN = 8	TN = 5

Confusion Matrix: isomiR	
TP = 82	FP = 3
FN = 4	TN = 6

Table S2: Selected miRNAs and isomiRs used in Decision Tree building. The 3 miRNAs and 4 isomiRs selected by the best and second-best models with the confusion matrix obtained from each prediction (P stands for positives, which are responders in the context of this study, N are negatives).

**Features considered by CART**	**Median MCC**	**Median AUC**
Clinical data	0.123	0.565
miRNA	0.322	0.643
isomiR	0.557	0.804
miRNA+ Clinical data	0.40	0.731
isomiR+Clinical data	0.549	0.781
miRNA+isomiR	0.22	0.62
All profiles	-0.016	0.491

Table S3: Comparison of the predictive performance of CART models trained on predictive molecular profiles only with those integrating other data modalities. The best and second-best models' predictive performance was compared with those combining other data, including clinical data (gender, age, tumor stage and tumor grade); merging the two predictive profiles (miRNA & isomiR), and integrating the eight molecular profiles considered in this study.

**MCC**	**HER2**	**miRNA**	**isomiR**
Seed 1	0.14	0.264	0.526
Seed 2	0.14	0.22	0.592
Seed 3	0.14	0.394	0.557
Seed 4	0.14	0.386	0.557
Seed 5	0.14	0.322	0.373

**AUC**	**HER2**	**miRNA**	**isomiR**
Seed 1	0.57	0.632	0.798
Seed 2	0.57	0.62	0.81
Seed 3	0.57	0.731	0.804
Seed 4	0.57	0.693	0.804
Seed 5	0.57	0.643	0.725

Table S4: Comparison of five 10-fold CV CART models trained on miRNA and isomiR profiles with the prediction from HER2 (ERBB2) expression data. The best and second-best models' predictive performance was compared with those obtained from HER2 expression only.

Enrichment Pathway	Gene Size	# Gene Overlap	Expected Value	ER	p-value	FDR
ErbB Signaling Pathway	91	82	70.278	1.167	0.0010	0.0494
Endometrial cancer	63	58	48.654	1.192	0.0016	0.0494
DNA Damage Response [only Ataxia-Telangiectasia Mutated (ATM) dependent]	110	97	84.951	1.142	0.0021	0.0494
Chromosomal and microsatellite instability in colorectal cancer	73	66	56.377	1.171	0.0026	0.0494

*Expected value = gene set size / #reference * #input gene, ER= overlap/expected value.*
ER: enrichment ratio; FDR: false discovery rate,

Table S5: Enrichment analysis of 13,898 cumulative genes targeted by the identified miRNAs and isomiRs. The overall list (Table 1) obtained contains **13,898** genes targeted by all the miRNAs and the isomiRs predicted in our study, out of which **12,967** were unambiguously mapped to the reference genome (genome protein-coding), and **930** genes could not be mapped to reference. Four (4) pathways are significantly (FDR < 0.05) enriched with our predicted genes; the number of genes that are associated with each of the pathways are presented in this table. For example, out of the total of 110 genes that are found in the DNA damage response pathway, 97 were targeted by our predicted miRNAs. The gene ontology for these mapped genes is presented in Figure S5A

Enrichment Pathway	Gene Size	#Gene overlap	Expected value	ER	p-value	FDR	Gene Overlap
Non-small cell lung cancer	72	6	0.822	7.302	0.00009919	0.00561	ERBB2;AKT1;PIK3CA;RB1; TP53;CASP8
Breast cancer pathway	154	8	1.758	4.552	0.00001596	0.00561	ERBB2;ESR1;AKT1;PIK3CA;RB1; TP53;BRCA2;BRCA1
Tumor suppressor activity of SMARCB1	31	4	0.354	11.305	0.0003235	0.00561	RB1;ARID1B;SMARCD1;ARID1A
Pancreatic adenocarcinoma pathway	89	6	1.016	5.907	0.000326	0.00561	ERBB2;AKT1;PIK3CA;RB1;TP53; BRCA2
ErbB Signaling Pathway	91	6	1.039	5.777	0.000369	0.00561	MAP2K4;ERBB2;AKT1;PIK3CA; CDKN1B;TP53
Endometrial cancer	63	5	0.719	6.954	0.000534	0.00677	ERBB2;AKT1;PIK3CA;TP53; CDH1
DNA Damage Response	68	5	0.776	6.443	0.000764	0.00829	RB1;CDKN1B;TP53;BRCA1;CASP8
Bladder Cancer	40	4	0.457	8.762	0.000879	0.00835	ERBB2;RB1;TP53;CDH1
DNA Damage Response (only ATM dependent)	110	6	1.255	4.779	0.00103	0.00874	ERBB2;AKT1;PIK3CA;CDKN1B; TP53;MAP3K1
Apoptosis	84	5	0.959	5.215	0.002	0.0153	MAP2K4;AKT1;TP53;MAP3K1; CASP8

ER: enrichment ratio; FDR: false discovery rate

Table S6: Enrichment pathway for the 23 selected BC-associated genes among the genes predicted to be targeted by the predictive miRNAs. Enrichment analysis of the 23 potential breast cancer target genes filtered from the cumulative 13,898 predicted genes according to mutational breast cancer driver genes reported in Integrative OncoGenomics (IntOGen)[1] and Catalogue Of Somatic Mutations In Cancer (COSMIC)[2,3] databases revealed ten (10) significantly (p<0.01 & FDR<0.05) enriched cancer-associated pathways.

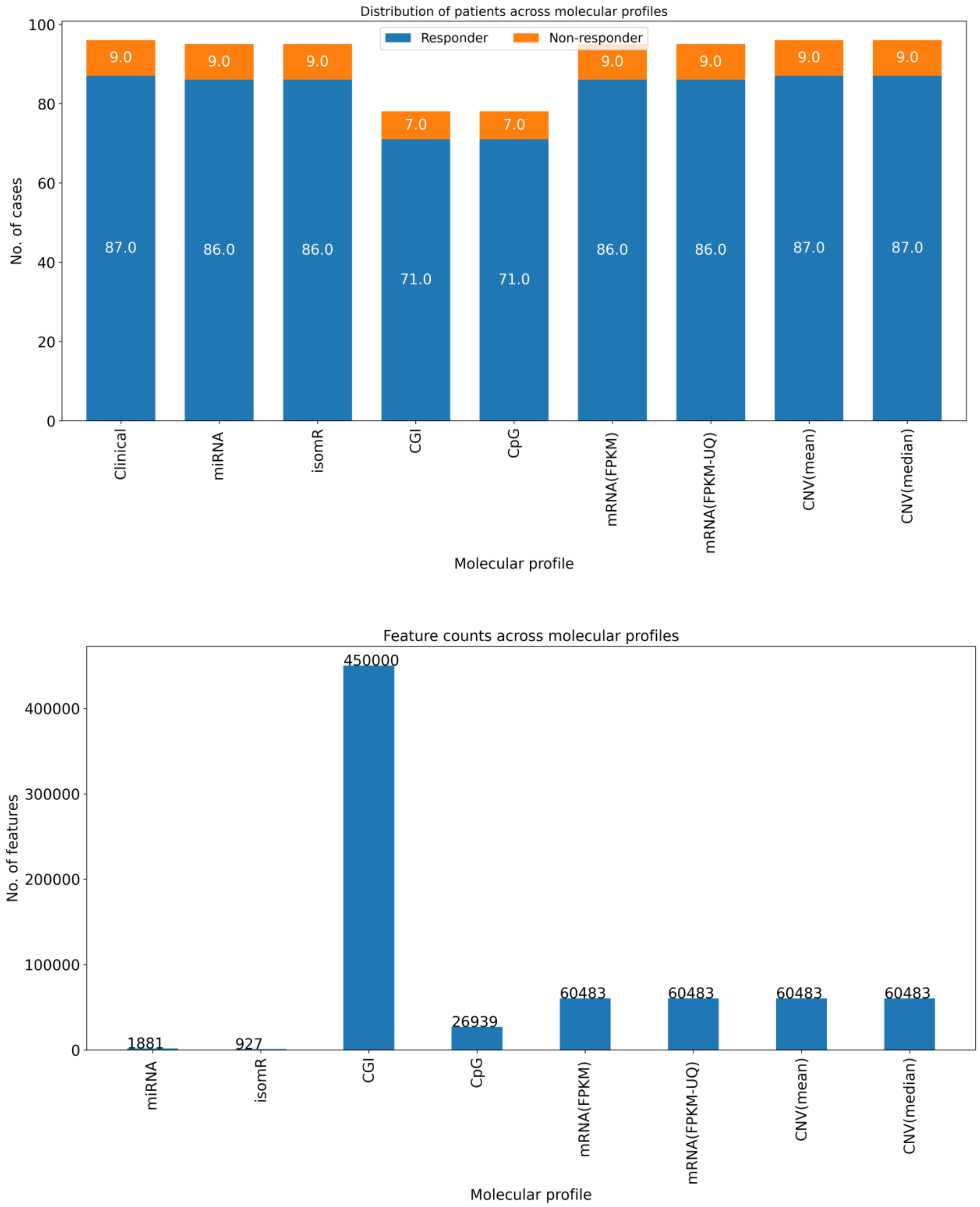

Figure S1: Patients' distribution across clinical and molecular profiles datasets: Bar charts presenting the curated clinical and molecular profiling datasets for the BC patients treated with doxorubicin-containing regimens for the GDC-TCGA project. The number of BC patients considered per each profiling data sets for predicting patients' response to doxorubicin treatment (top) and the number of features available per dataset (bottom) are shown.

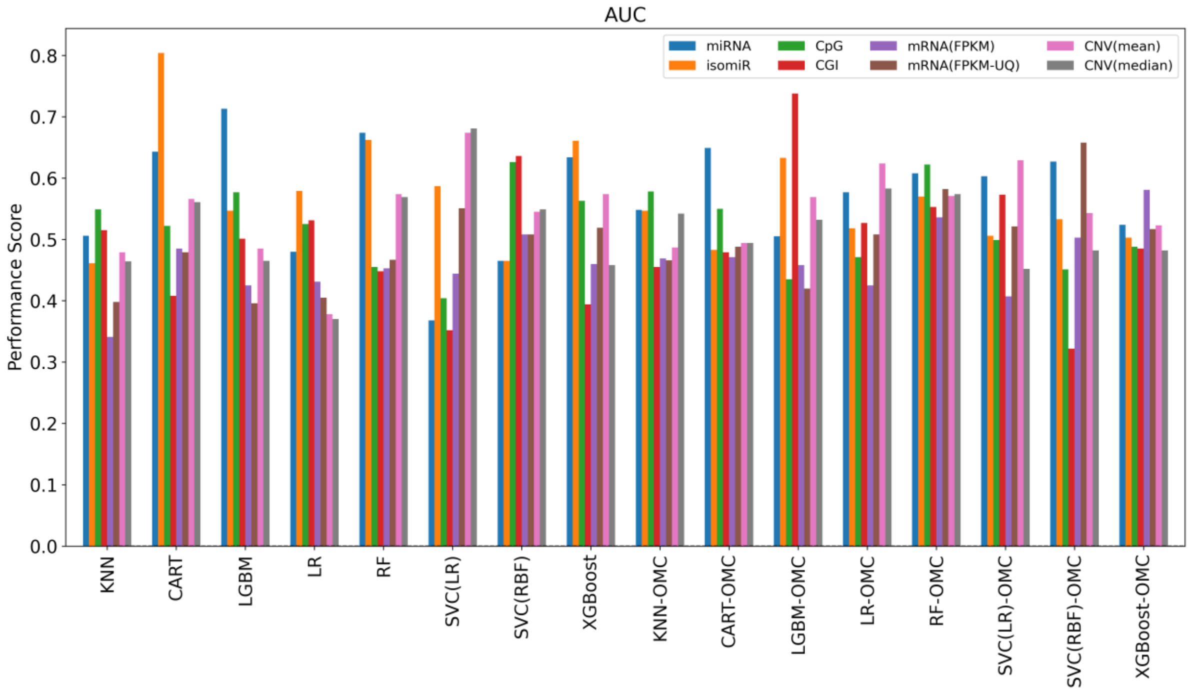
AUC
Performance Score
miRNA
isomiR
CpG
CGI
mRNA(FPKM)
mRNA(FPKM-UQ)
CNV(mean)
CNV(median)
KNN
CART
LGBM
LR
RF
SVC(LR)
SVC(RBF)
XGBoost
KNN-OMC
CART-OMC
LGBM-OMC
LR-OMC
RF-OMC
SVC(LR)-OMC
SVC(RBF)-OMC
XGBoost-OMC

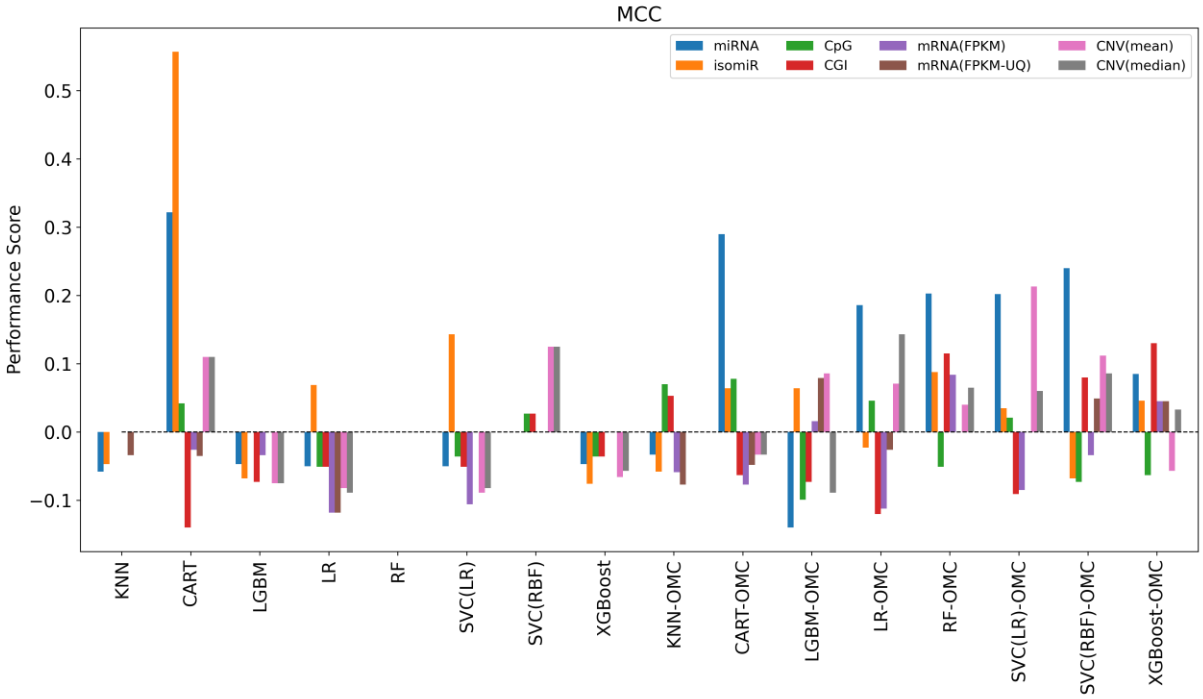
MCC
Performance Score
miRNA
isomiR
CpG
CGI
mRNA(FPKM)
mRNA(FPKM-UQ)
CNV(mean)
CNV(median)
KNN
CART
LGBM
LR
RF
SVC(LR)
SVC(RBF)
XGBoost
KNN-OMC
CART-OMC
LGBM-OMC
LR-OMC
RF-OMC
SVC(LR)-OMC
SVC(RBF)-OMC
XGBoost-OMC

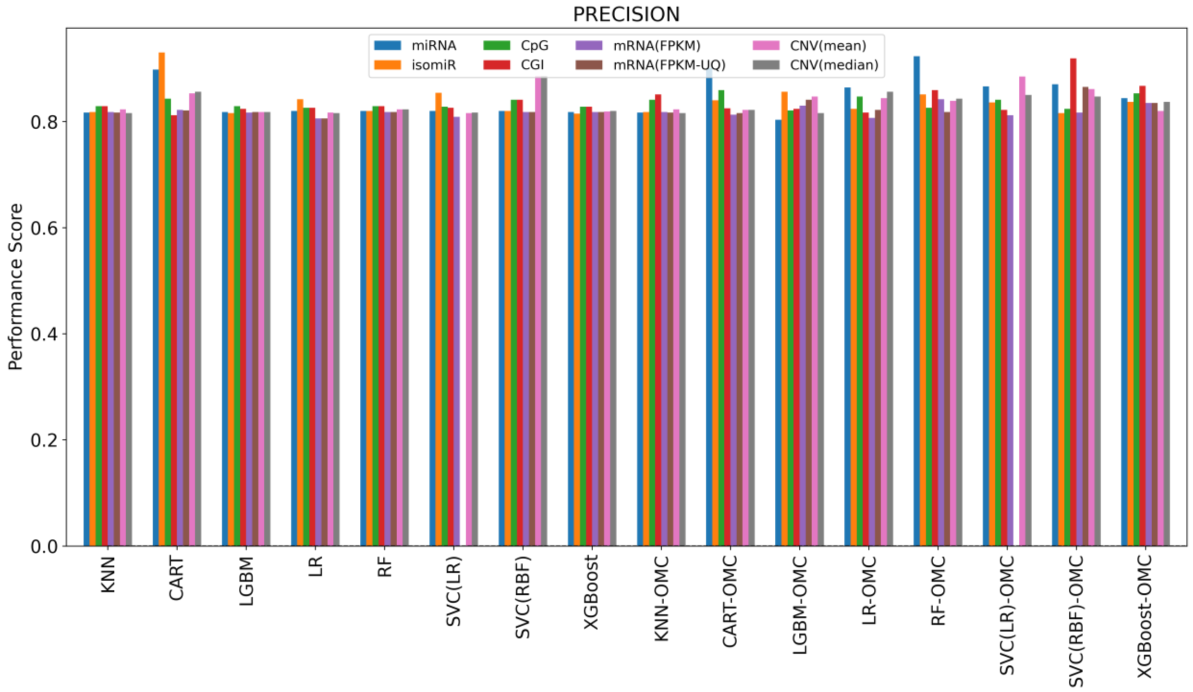

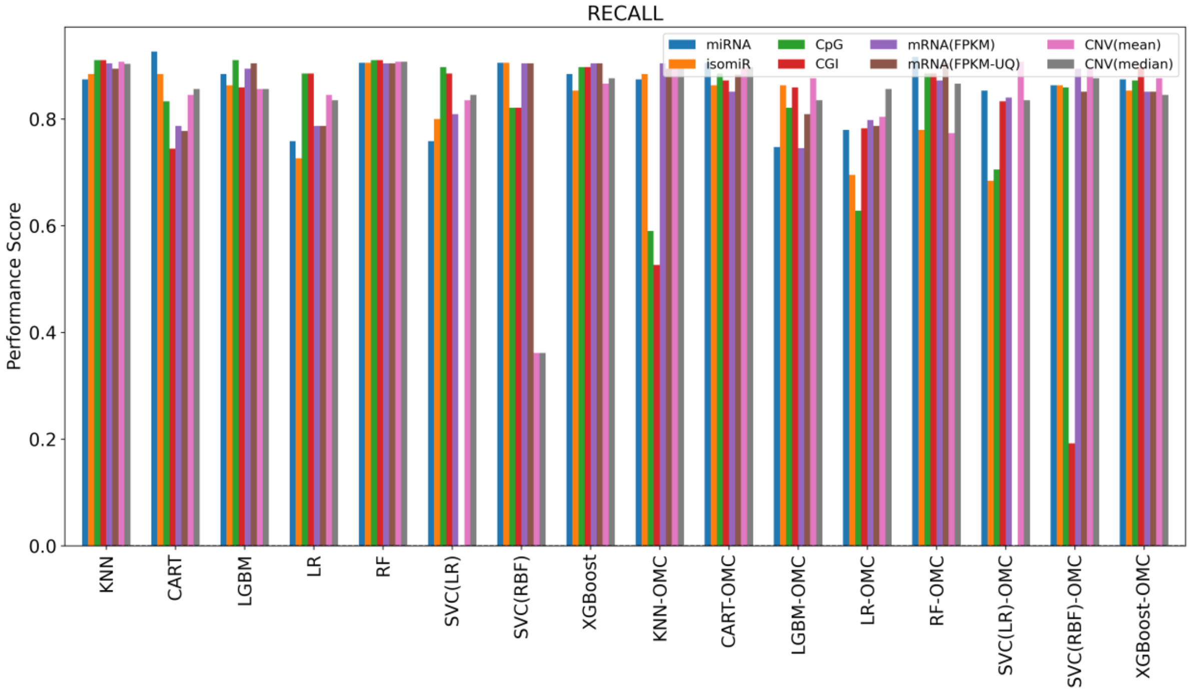

Figure S2 (Related to Figure 2): Median scores of various evaluation metrics across models and molecular profiles. Bar charts presenting the median AUC-ROC, MCC, precision and recall scores obtained across five runs of 10-fold CV implemented on each of the molecular profiles considered in this study. The first 8 algorithms on the left were ran without OMC whereas the rest of algorithms (the other 8 on the right) were ran with OMC to search for a small subset of features facilitating the classification of patients. 10-by-10 nested-CV runs were carried out five times, each time with a different random seed

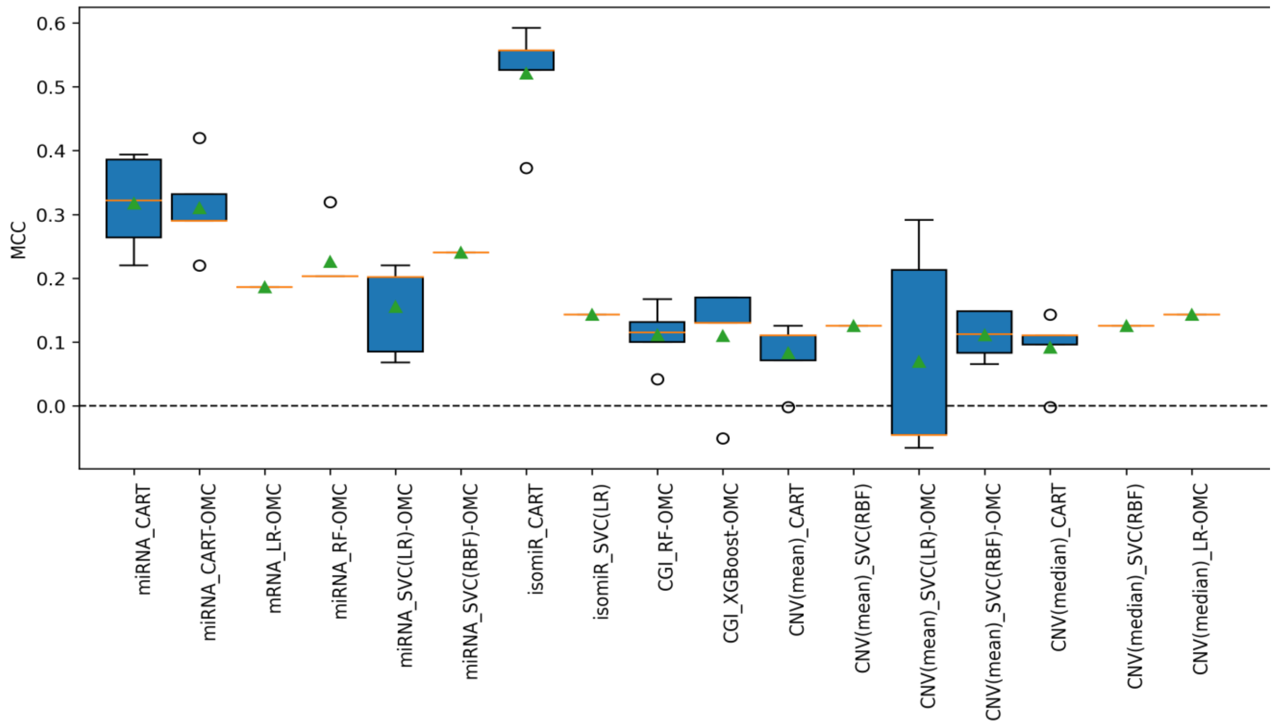

Figure S3: MCC variability across the 5 random seeds for each of the models with a median MCC (mMCC) of at least 0.1. Among the 7 models with mMCC of at least 0.2, 5 employed OMC; miRNA and their isoforms are the most informative features to predict the response of BC patients treated with doxorubicin-containing regimens, with isomiR features and the CART algorithm being the most predictive model (mMCC of 0.56).

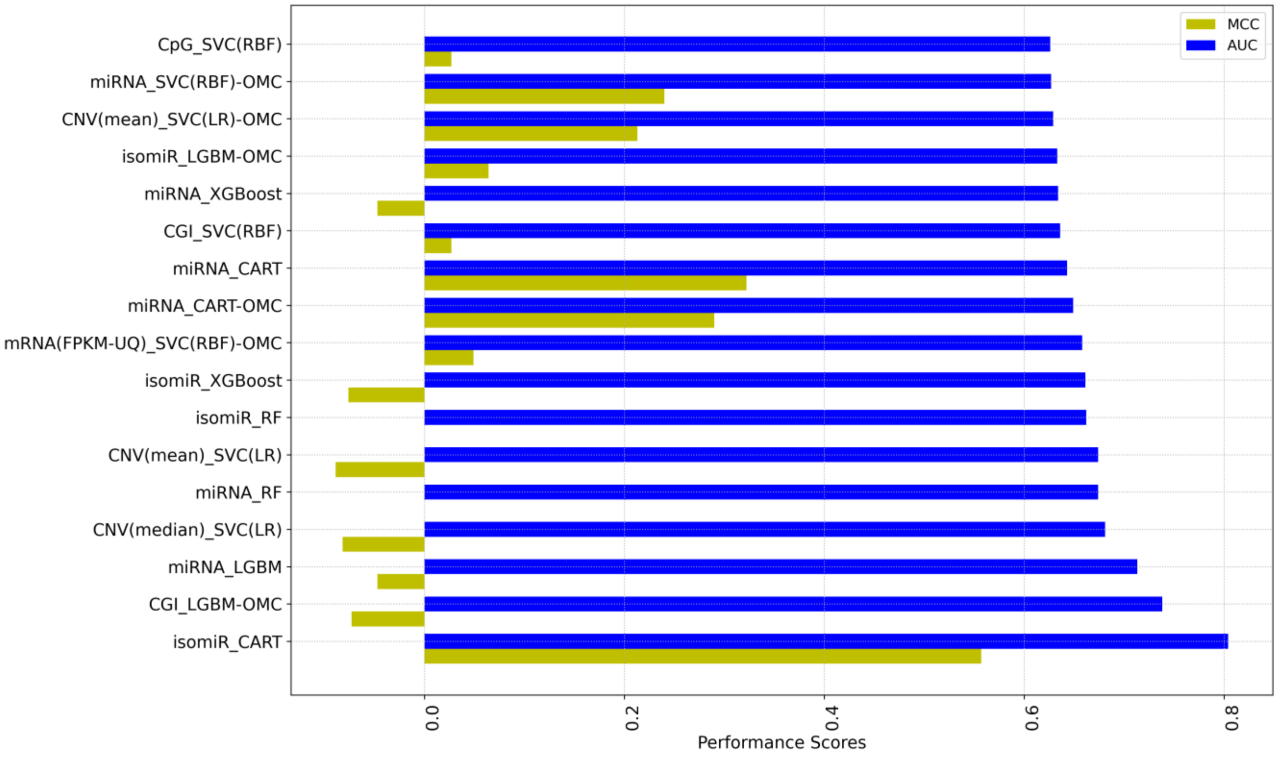

Figure S4: Bar chart presenting the median MCC and the corresponding AUC scores of the 17 models with the highest AUC: Models with good MCC also have good AUC. By contrast, many models with good AUC have poor MCC (e.g. miRNA_LGBM). The latter models misclassified all the patients in the minority class (non-responders), which means that model detects no true negatives, thus leading to near-zero MCC values. This further shows that AUC is an unsuitable metric for evaluating the imbalanced dataset, making MCC a better choice for this type of problem.

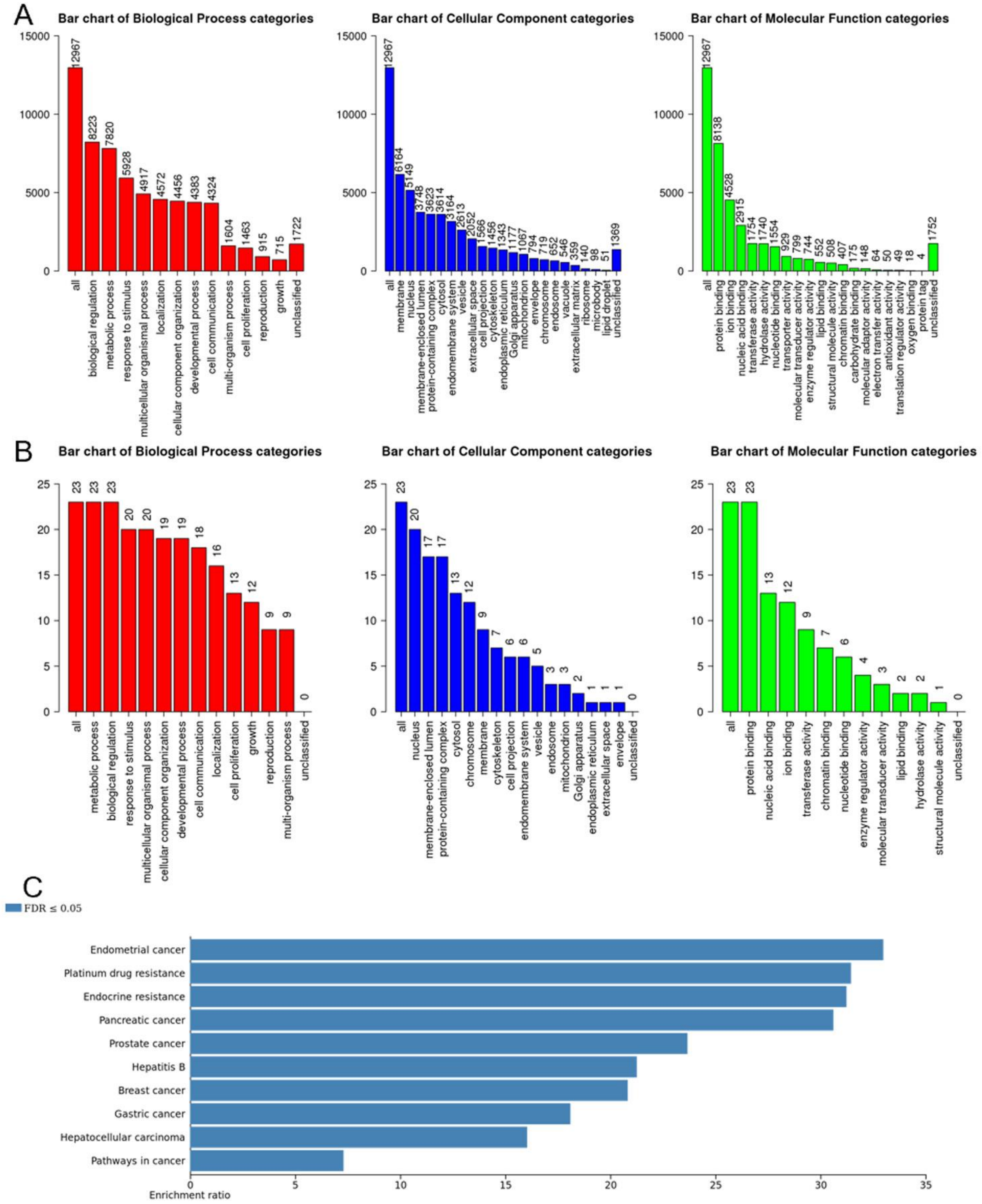

Figure S5: KEGG Enrichment analysis: Bar charts presenting the number of genes involved in various biological processes, their cellular locations and molecular functions; (A) for all the 13,898 overall unique genes predicted to be targeted by the selected miRNAs and its isomiR (B) for 23 overlapping genes between the two databases (COSMIC and *IntOGen*) employed in filtering cancer-associated genes targeted by our predictive miRNAs and isomiRs. (C) Over Representation Analysis (ORA) of the 23 overlapping genes using KEGG repository. Dysregulation of any of the miRNAs predicted in this study, could lead to the dysregulation of their target genes and the pathways in which they are involved (presented above) which may consequently result in doxorubicin resistance in BC patients. In BC, oncomiRs have been shown to induce chemoresistance via different mechanisms, including regulating vital resistance-associated proteins, repression or silencing of genes that induce cancer apoptosis or cell survival processes[4–6].

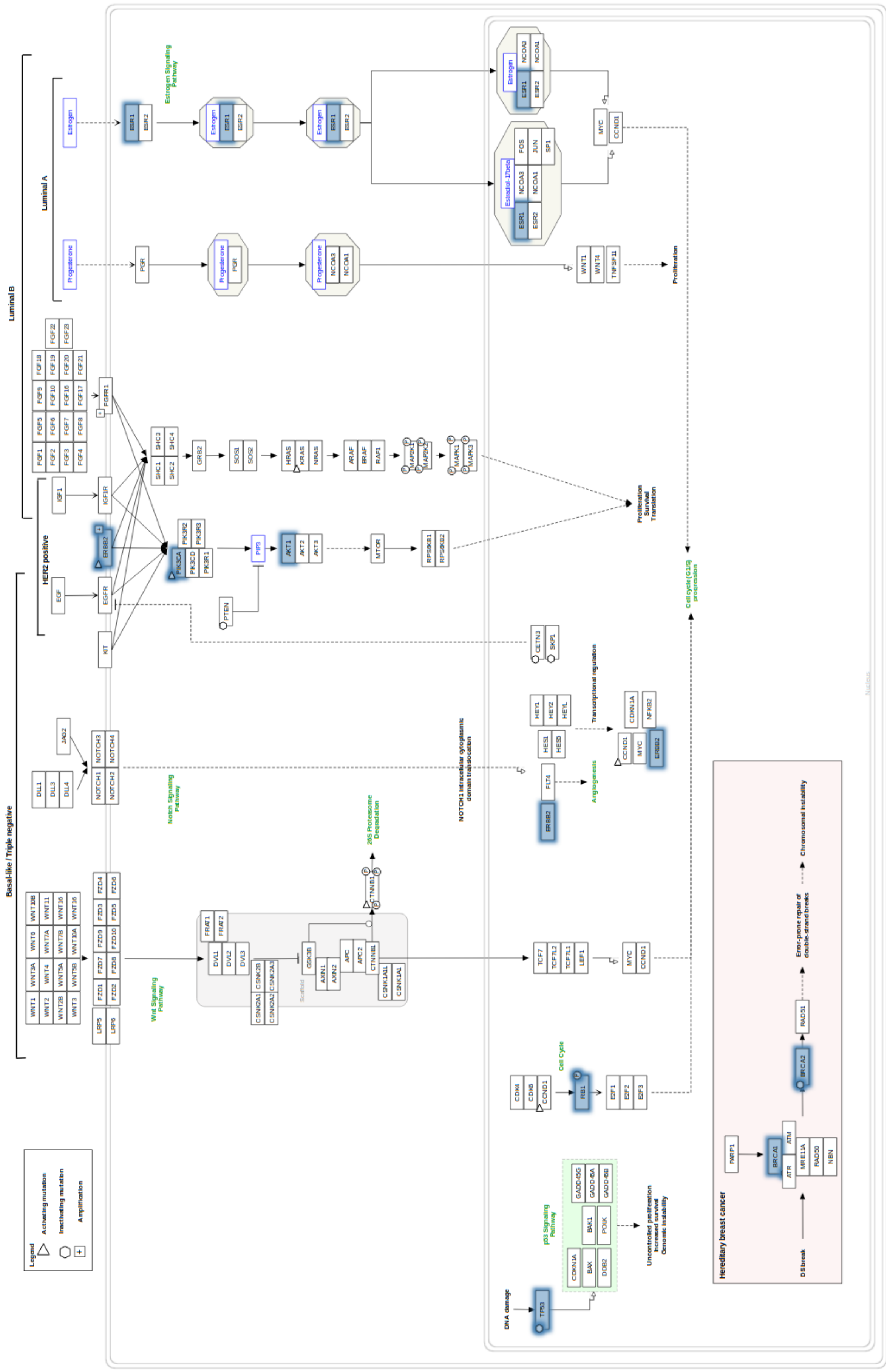
Luminal B
Luminal A
HER2 positive
Basal-like / Triple negative
Hereditary breast cancer
Legend
Activating mutation
Inactivating mutation
Amplification

Figure S6: WikiPathways cancer analysis: Schematic presentation of the interactions between the predicted target genes and the rest of genes associated with breast cancer pathways. The 8 genes highlighted in blue represent genes targeted by the predicted miRNAs. Dysregulation of the genes could promote resistance to doxorubicin.

References

1. Martínez-Jiménez, F., Muiños, F., Sentís, I., Deu-Pons, J., Reyes-Salazar, I., Arnedo-Pac, C., Mularoni, L., Pich, O., Bonet, J., Kranas, H., Gonzalez-Perez, A., and Lopez-Bigas, N. (2020) A compendium of mutational cancer driver genes. *Nat. Rev. Cancer*, **20** (10), 555–572.
2. Sondka, Z., Bamford, S., Cole, C.G., Ward, S.A., Dunham, I., and Forbes, S.A. (2018) The COSMIC Cancer Gene Census: describing genetic dysfunction across all human cancers. *Nat. Rev. Cancer*, **18** (11), 696–705.
3. Tate, J.G., Bamford, S., Jubb, H.C., Sondka, Z., Beare, D.M., Bindal, N., Boutselakis, H., Cole, C.G., Creatore, C., Dawson, E., Fish, P., Harsha, B., Hathaway, C., Jupe, S.C., Kok, C.Y., Noble, K., Ponting, L., Ramshaw, C.C., Rye, C.E., Speedy, H.E., Stefancsik, R., Thompson, S.L., Wang, S., Ward, S., Campbell, P.J., and Forbes, S.A. (2018) COSMIC: The Catalogue Of Somatic Mutations In Cancer. *Nucleic Acids Res.*, **47** (D1), D941--D947.
4. Zhang, J.X., Chen, Z.H., Chen, D.L., Tian, X.P., Wang, C.Y., Zhou, Z.W., Gao, Y., Xu, Y., Chen, C., Zheng, Z.S., Weng, H.W., Ye, S., Kuang, M., Xie, D., and Peng, S. (2018) LINC01410-miR-532-NCF2-NF-kB feedback loop promotes gastric cancer angiogenesis and metastasis. *Oncogene*, **37** (20), 2660–2675.
5. Zhang, Z., Li, Z., Li, Y., and Zang, A. (2014) MicroRNA and signaling pathways in gastric cancer. *Cancer Gene Ther.*, **21** (8), 305–316.
6. Mulrane, L., McGee, S.F., Gallagher, W.M., and O’Connor, D.P. (2013) miRNA dysregulation in breast cancer. *Cancer Res.*, **73** (22), 6554–6562.
